# The longitudinal loss of islet autoantibody responses from diagnosis of type 1 diabetes occurs progressively over follow-up and is determined by low autoantibody titres, early-onset, and genetic variants

**DOI:** 10.1093/cei/uxac087

**Published:** 2022-10-01

**Authors:** C L Williams, R Fareed, G L M Mortimer, R J Aitken, I V Wilson, G George, K M Gillespie, A J K Williams, The BOX Study Group, Chitrabhanu Ballav, Atanu Dutta, Michelle Russell-Taylor, Rachel Besser, James Bursell, Shanthi Chandran, Sejal Patel, Anne Smith, Manohara Kenchaiah, Gomathi Margabanthu, Foteini Kavvoura, Chandan Yaliwal, A E Long

**Affiliations:** Diabetes and Metabolism, Translational Health Sciences, Bristol Medical School, University of Bristol, Level 2, Learning and Research Building, Southmead Hospital, Bristol BS10 5NB, UK; Diabetes and Metabolism, Translational Health Sciences, Bristol Medical School, University of Bristol, Level 2, Learning and Research Building, Southmead Hospital, Bristol BS10 5NB, UK; Diabetes and Metabolism, Translational Health Sciences, Bristol Medical School, University of Bristol, Level 2, Learning and Research Building, Southmead Hospital, Bristol BS10 5NB, UK; Diabetes and Metabolism, Translational Health Sciences, Bristol Medical School, University of Bristol, Level 2, Learning and Research Building, Southmead Hospital, Bristol BS10 5NB, UK; Diabetes and Metabolism, Translational Health Sciences, Bristol Medical School, University of Bristol, Level 2, Learning and Research Building, Southmead Hospital, Bristol BS10 5NB, UK; Diabetes and Metabolism, Translational Health Sciences, Bristol Medical School, University of Bristol, Level 2, Learning and Research Building, Southmead Hospital, Bristol BS10 5NB, UK; Diabetes and Metabolism, Translational Health Sciences, Bristol Medical School, University of Bristol, Level 2, Learning and Research Building, Southmead Hospital, Bristol BS10 5NB, UK; Diabetes and Metabolism, Translational Health Sciences, Bristol Medical School, University of Bristol, Level 2, Learning and Research Building, Southmead Hospital, Bristol BS10 5NB, UK; Bucks Healthcare Trust, UK; Bucks Healthcare Trust, UK; Bucks Healthcare Trust, UK; Oxford University Hospitals Trust UK, UK; Milton Keynes University Hospital, UK; Milton Keynes University Hospital, UK; Wexham Park Hospital, UK; Northampton General Hospital, UK; Northampton General Hospital, UK; Kettering General Hospital, UK; Royal Berkshire Hospital, UK; Royal Berkshire Hospital, UK; Diabetes and Metabolism, Translational Health Sciences, Bristol Medical School, University of Bristol, Level 2, Learning and Research Building, Southmead Hospital, Bristol BS10 5NB, UK

**Keywords:** autoantibodies, autoimmunity, autoantibody loss, autoantibody specificity, islet autoimmunity, type 1 diabetes

## Abstract

The clinical usefulness of post-diagnosis islet autoantibody levels is unclear and factors that drive autoantibody persistence are poorly defined in type 1 diabetes (T1D). Our aim was to characterise the longitudinal loss of islet autoantibody responses after diagnosis in a large, prospectively sampled UK cohort. Participants with T1D [*n* = 577] providing a diagnosis sample [range −1.0 to 2.0 years] and at least one post-diagnosis sample (<32.0 years) were tested for autoantibodies to glutamate decarboxylase 65 (GADA), islet antigen-2 (IA-2A), and zinc transporter 8 (ZnT8A). Select HLA and non-HLA SNPs were considered. Non-genetic and genetic factors were assessed by multivariable logistic regression models for autoantibody positivity at initial sampling and autoantibody loss at final sampling. For GADA, IA-2A, and ZnT8A, 70.8%, 76.8%, and 40.1%, respectively, remained positive at the final sampling. Non-genetic predictors of autoantibody loss were low baseline autoantibody titres (*P* < 0.0001), longer diabetes duration (*P* < 0.0001), and age-at-onset under 8 years (*P* < 0.01–-0.05). Adjusting for non-genetic covariates, GADA loss was associated with low-risk HLA class II genotypes (*P* = 0.005), and SNPs associated with autoimmunity *RELA*/11q13 (*P* = 0.017), *LPP*/3q28 (*P* = 0.004), and negatively with *IFIH1*/2q24 (*P* = 0.018). IA-2A loss was not associated with genetic factors independent of other covariates, while ZnT8A loss was associated with the presence of HLA A*24 (*P* = 0.019) and weakly negatively with *RELA*/11q13 (*P* = 0.049). The largest longitudinal study of islet autoantibody responses from diagnosis of T1D shows that autoantibody loss is heterogeneous and influenced by low titres at onset, longer duration, earlier age-at-onset, and genetic variants. These data may inform clinical trials where post-diagnosis participants are recruited.

## Teaser

Loss of islet autoantibodies after diabetes onset is heterogeneous but influenced by non-genetic and genetic factors.

## Graphical Abstract

**Graphical Abstract:**
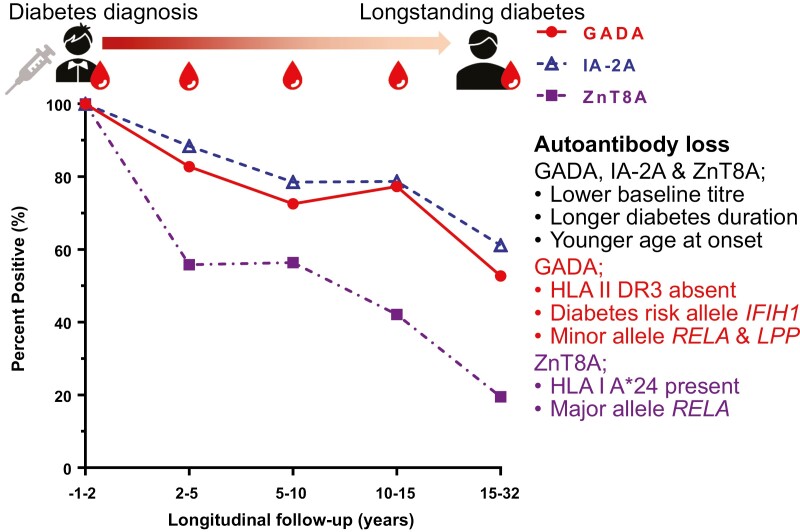

## Introduction

Four major type 1 diabetes (T1D)-associated autoantibodies to insulin (IAA), glutamate decarboxylase 65 (GADA), islet antigen-2 (IA-2A), and zinc transporter 8 (ZnT8A) remain primary biomarkers for predicting future diabetes and are distinctive clinical features of islet autoimmunity. At the onset of clinically diagnosed T1D, >90% of people are positive for at least one of these autoantibodies [1, 2]. The appearances of specific autoantibodies during the preclinical phase of diabetes and those present at onset are associated with HLA class II genotypes, age-at-onset, gender [3–6] and to a lesser degree, with HLA class I and non-HLA genotypes [7–9].

Studies of autoantibody prevalence after T1D diagnosis spanning 12–56 years (diagnosed between 0 and 56 years of age) generally report GADA as the most frequently detected autoantibody, followed by IA-2A and ZnT8A (where tested) [10]. These studies also suggest that autoantibody prevalence after T1D onset shows associations with a range of non-genetic (autoantibody specificity, number of autoantibodies, gender, age-at-onset, and diabetes duration at time of autoantibody detection) and genetic (HLA and non-HLA) factors [11–14]. Factors that influence the development of autoantibodies during the T1D prodrome may therefore, continue to drive humoral responses after T1D onset. However, these findings are often from small and largely cross-sectional cohorts, which differ by study design parameters (population, ethnicity, age-at-onset, duration of diabetes at the time of autoantibody detection, panel of autoantibodies tested, and autoantibody detection methods) [10]. Hence, most of these studies cannot account for baseline autoantibody presence, titre, or profile around T1D onset to differentiate prevalence from the persistence of humoral responses. Few longitudinal studies measured all major biochemical islet autoantibodies around onset and follow-up and these comprised of ~100 individuals diagnosed in childhood to young adulthood (<22 years) with follow-up of ≥10 years, which limited investigating the breadth of the humoral response [15, 16].

Understanding why autoantibody positivity is lost or maintained post-diagnosis may provide insights into the ongoing loss of beta-cells and insulin secretion. Autoantibody positivity is inversely correlated with diabetes duration but does not appear to strongly correlate with C-peptide, a by-product of insulin synthesis and a biomarker for exogenous insulin requirement [12, 15, 17–20]. However, these studies report conflicting findings, which may in part, be due to the variable rate of C-peptide decline after diagnosis or the nature of cross-sectional sampling [21]. Consequently, the relationship between residual beta-cells (providing antigen stimulus to the immune system) or other drivers for continued islet autoantibody production remains unclear. Due to the lack of longitudinal sampling and limited clinical data in many cohorts with post-diagnosis autoantibody data, the biological correlates and clinical usefulness of autoantibody responses after diagnosis are also understudied. We therefore sought to investigate longitudinal autoantibody profiles and whether the non-genetic and genetic determinants were different between autoantibody prevalence close to diagnosis and islet autoantibody loss using data from T1D subjects that provided both baseline (with confirmed autoantibody positivity status) and longitudinal follow-up samples in a large, long-running population-based UK cohort.

## Research design and methods

### Cohort description

The population-based Bart’s-Oxford (BOX) family study, established in 1985, has recruited and prospectively followed individuals with newly diagnosed T1D under 21 years (with confirmed initiation of exogenous insulin treatment) and their first-degree relatives within the UK’s former Oxford Regional Health Authority. The BOX study is currently approved by the South Central—Oxford C. National Research Ethics Committee. Participants provided informed, written consent and the study was performed according to the principles of the Declaration of Helsinki. All BOX participants that had a serum sample taken close to T1D onset [median 0.11 years (range −0.86 to 1.98), *n* = 419 <0.5 years] and at least one longitudinal serum sample at follow-up [median 7.3 years (range 2.0–32.0)] were selected and tested for GADA, IA-2A, and ZnT8A; multiple longitudinal samples were tested until autoantibody negativity was determined.

A total of 577 [320 male (55.5%); median age-at-onset 10.74 years (range 0.74–54.6)] autoantibody positive individuals at onset were identified. Data for IAA were available from 238 individuals (41.2%) and considered in select analyses where the onset sample was taken within 2 weeks of diagnosis before exogenous insulin treatment. Overall, 290 (50.3%), 183 (31.7%), 87 (15.1%), and 17 (2.9%) individuals provided 1, 2, 3, and 4 follow-up samples, respectively; combinations of samples available for autoantibody detection across categories of follow-up are detailed in Supplementary Table S1. The distribution of longitudinal serum samples available for autoantibody detection across time categories of follow-up is detailed in Supplementary Fig. S1. Characteristics of the cohort with available data for variables investigated are detailed in Table 1.

**Table 1:** *Cohort description and all variables investigated for association with autoantibody loss for GADA, IA-2A, and ZnT8A after onset of type 1 diabetes.* For Non-HLA SNPS, asterisks identify minor allele.

Variable	Number (%)
**Gender (*n* = 577)**	
Male	320 (55.5)
Female	257 (44.5)
**Age at onset (*n* = 577)**	
0.74–7.52 years	144 (25.0)
7.52–10.73 years	144 (25.0)
10.73–13.76 years	144 (25.0)
>13.76–54.6 years	145 (25.0)
**Autoantibody (*n* = 577)**	
IAA (*n* = 238)	171 (71.8)
GADA	487 (84.4)
IA-2A	452 (78.3)
ZnT8A	395 (68.5)
ZnT8RA	342 (59.3)
ZnT8WA	297 (51.5)
**HLA Class II (*n* = 501)**	
High (DR3-DQ2/DR4-DQ8)	159 (31.7)
Moderate (DQ2/DQ2, DQ8/DQ8, DQ2/X, DQ8/X)	269 (53.7)
Low (X/X, DQ6/X)	73 (14.6)
**HLA Class I**	
HLA-A*24 negative (*n* = 454)	376 (82.8)
HLA-B*18 negative (*n* = 417)	364 (87.3)
HLA-B*39 negative (*n* = 417)	385 (92.3)
**Non-HLA SNPs**	
*IFIH1* (*n* = 469)	
G	95 (41.6)
AG	215 (45.8)
A*	59 (12.6)
*RELA* (FIBP) (*n* = 432)	
C	280 (64.8)
CT	133 (30.8)
T*	19 (4.4)
*LPP* (*n* = 440)	
C	128 (29.1)
AC	213 (48.4)
A*	99 (22.5)
*FCRL3* (*n* = 442)	
G	159 (36.0)
AG	199 (45.0)
A*	84 (19.0)
*SLC30A8* (*n* = 383)	
CC	179 (46.7)
CT	168 (43.9)
TT*	36 (9.4)

### Autoantibody determination

Autoantibodies to GAD65 (aa1-585), IA-2ic (aa606-aa979), and ZnT8 [aa268-369; monomeric peptides encoding either arginine (R) or tryptophan (W) at aa325 to detect arginine-specific (ZnT8RA) and tryptophan-specific (ZnT8WA) ZnT8A] were detected by radioimmunoassays described previously [1, 22]. Logarithmic standard curves were used to determine units; GADA/IA-2A were expressed in Diabetic Kidney (DK) units/ml, and ZnT8A was expressed in arbitrary units (AU). Positivity thresholds were set at the percentiles of healthy controls for GADA (97th percentile of 1000 adults), IA-2A (98th of 500 adults), and ZnT8A (97.5th percentile of 523 schoolchildren) [23]. The methodology and threshold for IAA, described previously [24], were set at the 97.5th percentile of 2860 healthy schoolchildren. The sensitivity at 95% specificity of these assays was assessed in the 2020 Islet Autoantibody Standardisation Program: 78% GADA, 74% IA-2A, 70% ZnT8RA, 56% ZnT8WA, and 62% IAA. For the assessment of ZnT8A, the maximum ZnT8A result between ZnT8RA and ZnT8WA radioimmunoassays was used for analysis as responses were comparable over follow-up (data not shown).

### Genetic determination

All available DNA samples were extracted from whole blood or mouth swab samples and whole-genome amplified (Illustra GenomiPhi V2 DNA amplification kit; GE Healthcare). HLA class II alleles were determined in 501 participants using PCR sequence-specific primers (SSP) as described previously [25], and categorised into high [DR3-DQ2/DR4-DQ8; DRB1*03-DQA1*05:01-DQB1*02:01/DRB1*04-DQA1*03:01-DQB1*03:02 (reference variable)], moderate (*DQ8/DQ8, DQ8/X, DQ/X,* and *DQ2/DQ2*), and low risk (*X/X* and *DQ6/X*), where *X* refers to any other haplotype. Individuals with the protective *DQ6*/X genotype were categorised as low risk, and only present in four individuals (0.8% of 501). HLA class I genotypes for HLA-A*24 (*n* = 454), HLA-B*18 (*n* = 417), and HLA-B*39 (*n* = 417) determined by PCR-SSP as previously described [26], were coded as binary variables [positive/negative (reference variable)]. Non-HLA SNPs for *IFIH1*/2q24 (rs2111485 in LD with rs1990760, *n* = 469), *RELA*/11q13 (intron 4 of the *FIBP* gene; rs568617; *n* = 432), *LPP*/3q28 (rs1464510; *n* = 440), and *FCRL3*/1q23 (rs3761959; *n* = 442). These were chosen because of the strong effect size (OR > 1.25) for *RELA*, *LPP*, and *FCRL3*, and because *IFIH1* was associated with GADA and IA-2A, but in opposite directions, in analyses of islet autoantibodies after diagnosis by Brorsson *et al.* [11, 27]. For the assessment of ZnT8A, the *SLC30A8* SNP (rs13266634; *n* = 383) that determines two major epitopes of ZnT8A was considered [28]. The minor allele frequencies of all non-HLA SNPs considered in this cohort were of expected frequency within the general population and were coded as binary variables [minor/major (reference variable)] (Supplementary Table S2).

### Data transformation and statistical analysis

Data were analysed using SPSS (v. 27; IBM Corp., Armonk, N.Y., USA) and graphed using GraphPad PRISM (v.9.1.0; GraphPad Software, La Jolla California, USA). Proportions were compared using Chi-squared or Fisher’s exact tests where appropriate. Paired Wilcoxon tests were used to compare median autoantibody titre at onset and follow-up. Multivariable binary logistic regression was used to determine the influence of non-genetic [gender, age-at-onset (as quartiles, compared to the lowest quartile), time of sampling from onset (initial sampling (months) as a continuous variable and final sampling (years) as quartiles, compared to the lowest quartile), autoantibody titre at onset (units as quartiles, compared to highest quartile), number of- and combination of- autoantibodies at onset], and genetic covariates (categorical and binary) on autoantibody positivity at initial sampling and loss at final sampling. The Bonferroni correction was applied where applicable for multiple analyses (*P*_corr_). In all analyses, a two-tailed *P*-value < 0.05 was considered significant.

## Results

### Prevalence of autoantibody positivity at onset and longitudinal follow-up

**Figure 1:**
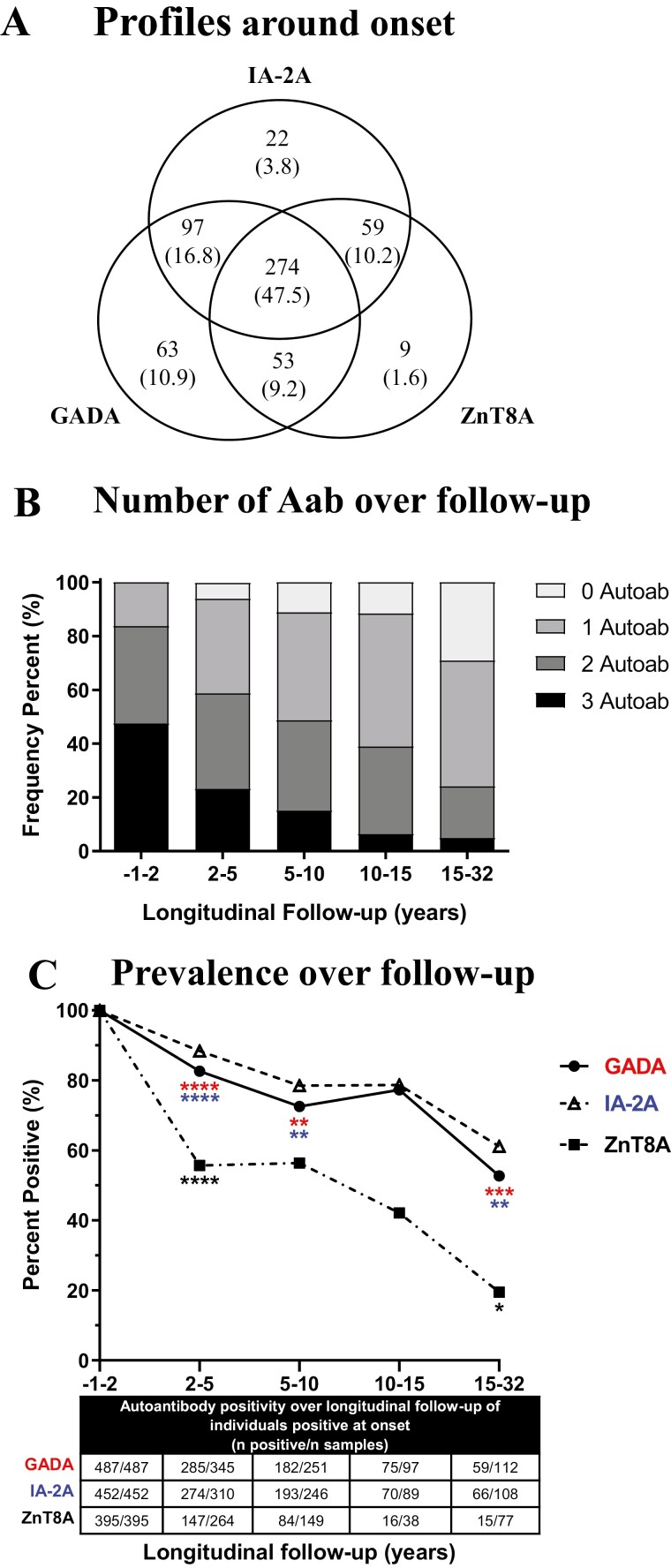
Prevalence of autoantibody positivity at type 1 diabetes onset and longitudinal follow-up. Autoantibody positivity profiles for GADA, IA-2A, and ZnT8A in 577 subjects sampled at onset of T1D (<2 years) that also had at least one follow-up serum sample (range 2.0–32.0 years). (a) Positivity for GADA, IA-2A, and ZnT8A. (b) The proportion of subjects positive for three and two autoantibodies decreased from onset (*P* < 0.0001) and the proportion of subjects positive for 1 autoantibody or 0 autoantibodies increased from onset (*P* < 0.0001). (c) Proportions of positivity for each autoantibody over longitudinal follow-up were compared to proportions at onset (all *P*_corr_ < 0.0001) and to the previous category of longitudinal follow-up (shown below data points); Red: GADA; Blue: IA-2A; Black: ZnT8A at significance with Bonferroni correction *P_corr_ < 0.05, **P_corr_ < 0.01, ***P_corr_ < 0.001 and ****P_corr_ < 0.0001.

Of 577 individuals prospectively followed, 84.4%, 78.3%, and 68.5% were positive at onset for GADA, IA-2A, and ZnT8A, respectively (Fig. 1A). Of the cohort, 16.3% had one, 36.2% had two, and 47.5% had three autoantibodies.

Individuals positive for ≥two autoantibodies decreased over longitudinal follow-up, whereas single autoantibody positive and autoantibody negative subjects increased (*P* < 0.0001; Fig. 1B). The proportion of individuals that lost at least one autoantibody, which was present at diagnosis, increased over follow-up (45.7% 2–5 years, 61.5% 5–10 years, 74.4% 10–15 years, and 83.0% 15–32 years). Profiles containing GADA and/or IA-2A were more frequent over follow-up than those containing ZnT8A (Supplementary Fig. S2).

Longitudinal autoantibody positivity showed distinct patterns according to antigen specificity. ZnT8A positivity was lost more rapidly than GADA and IA-2A whereas, IA-2A positivity was lost at a rate comparable with GADA over follow-up (Fig. 1C). In subjects positive for ZnT8A at onset (395/577; 68.5%;), only 40.1% (150/374 with complete data) remained positive at final sampling [median 6.1 years (range 2–32)], with the greatest proportionate loss occurring within 5 years of onset (44.2%, *P*_corr_ < 0.0001) and an overall loss of 80.5% at a diabetes duration ≥15 years (*P*_corr_ < 0.05 compared with 10–15 years). In contrast, in subjects positive for GADA at onset (487/577; 84.4%), 70.8% (340/480 with complete data) remained positive at final sampling [median 7.9 years (range 2–32)], with only 17.3% loss within 5 years of onset (*P*_corr_< 0.0001) and an overall loss of 47.3% at a diabetes duration ≥15 years (*P*_corr_<0.001 compared with 10–15 years). Comparable to GADA, in subjects positive for IA-2A at onset (452/577; 78.3%), 76.8% (347/452) remained positive at final sampling [median 8.0 years (range 2–32)], with only 11.6% loss occurring within 5 years of onset (*P*_corr_ < 0.0001) and an overall loss of 38.9% at a diabetes duration ≥15 years (*P*_corr_ < 0.01 compared with 10–15 years).

### Patterns in autoantibody titre over longitudinal follow-up

In accordance with decreasing autoantibody prevalence, the median autoantibody titre for GADA, IA-2A, and ZnT8A also decreased as a function of increasing diabetes duration (*P* < 0.0001) but high GADA and IA-2A titres were still observed at ≥15 years diabetes duration, which was rare for ZnT8A (Supplementary Fig. S3). Across a range of baseline titres, longitudinal autoantibody titres progressively decreased over follow-up in most subjects [GADA: 365/487 (74.9%); IA-2A: 407/452 (90.0%); ZnT8A: 389/395 (98.5%)]. A minority of GADA and IA-2A positive subjects had higher autoantibody titres in at least one follow-up sample compared to onset [GADA: *n* = 68 (14.0%); IA-2A: *n* = 25 (5.5%)] and/or had evidence of waxing–waning patterns of differing magnitudes from onset over at least two follow-up samples [GADA: *n* = 69 (14.2%); IA-2A: *n* = 26 (5.8%)]. However, only 6 (1.5%) ZnT8A positive subjects had a higher ZnT8A titre in at least 1 follow-up sample compared to onset, and there was no evidence of waxing–waning patterns (data not shown). Given the testing strategy, the re-emergence or seroconversion of additional autoantibodies after onset cannot be evaluated.

### Non-genetic associations of autoantibody positivity at initial sampling and autoantibody loss at final sampling

#### Initial sampling

Non-genetic factors previously associated with positivity for GADA, IA-2A, and ZnT8A at onset were confirmed in this cohort in adjusted multivariable models (Supplementary Fig. S4 and Supplementary Fig. S5). GADA and IA-2A positivity was associated with an older age-at-onset (>11 years and >14 years, respectively, *P* < 0.0001–0.001), whereas ZnT8A positivity was most common at diagnosis between 8 and 11 years old and IAA positivity was negatively associated with an older age-at-onset >11 years (*P* = 0.001–0.023). Females were more likely to have GADA (*P* = 0.026). Positivity for IA-2A at onset was associated with ZnT8A (*P* = 4 × 10^−6^) and IAA (*P* = 0.003) and ZnT8A positivity at onset was associated with IA-2A (*P* = 5 × 10^−6^) and weakly with IAA (*P* = 0.045) whereas, GADA positivity at onset was not associated with other autoantibodies. An increase in initial sampling (months) from onset was only negatively associated with ZnT8A positivity (*P* = 0.009), suggesting that loss of ZnT8A can occur within months from onset.

#### Final sampling

**Figure 2:**
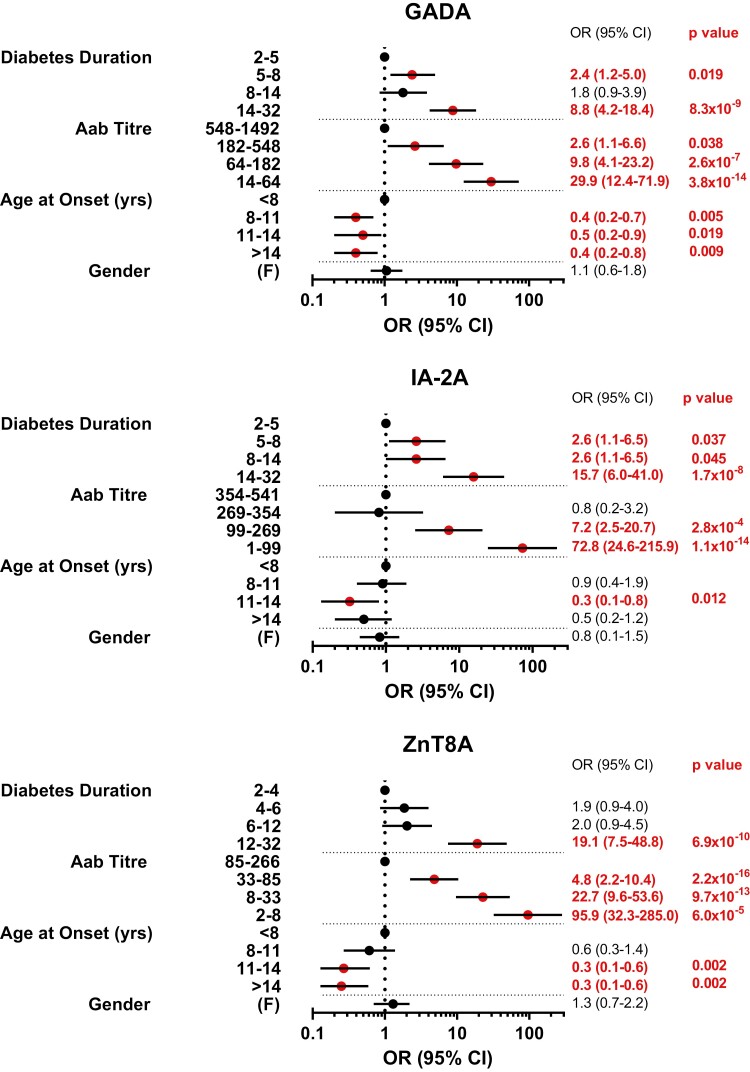
Non-genetic associations of autoantibody loss for GADA, IA-2A, and ZnT8A. OR: odds ratio; 95% CI: confidence interval; OR of 1 denotes the reference category of variable; ORs over 1 favours autoantibody loss; red dots and text denote alpha significance (<0.05). ORs, CIs, and *P* values were calculated from multivariable logistic regression models for autoantibody loss at final sampling time (years) adjusted for all non-genetic covariates [gender, age at onset, quartile of baseline autoantibody titre at onset, and quartile of disease duration from onset (years) at final sampling]. This multivariable non-genetic model was used as a baseline model for further analysis.

Multivariable analysis on the loss of GADA, IA-2A, and ZnT8A responses at final follow-up adjusted for all non-genetic covariates are detailed in Fig. 2. As expected, the quartile of diabetes duration at final follow-up (years) was associated with autoantibody loss to differing degrees (OR range 1.8–19.1; *P* < 0.0001–0.05) and was included in all multivariable analyses. Gender was not associated with autoantibody responses at final follow-up independent of other covariates (*P* > 0.05). Additionally, there was also no evidence of an interaction between baseline age and autoantibody titre on autoantibody loss at final sampling (*P* > 0.05) and was not included in multivariable analysis.

### Autoantibody titre at onset

**Figure 3:**
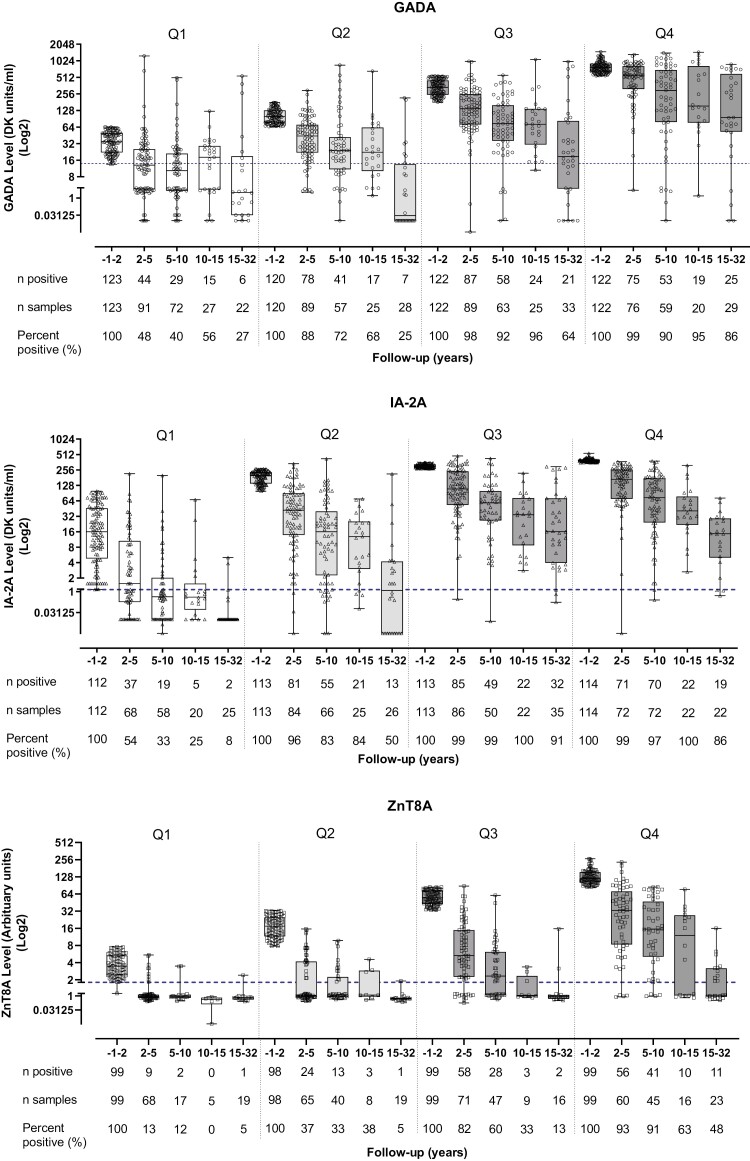
Longitudinal GADA, IA-2A, and ZnT8A titres according to the quartile of titre present at type 1 diabetes onset. The prevalence of longitudinal autoantibody positivity in all autoantibody responses was higher in subjects with high quartiles of baseline autoantibody titre (*P* < 0.0001–0.05). Independent of baseline titre, longitudinal autoantibody titres sequentially decreased over follow-up in most subjects [GADA: 403/487 (82.8%); IA-2A: 423/452 (93.6%); ZnT8A: 389/395 (98.5%)].

Multivariable analysis confirmed that lower quartiles of autoantibody titres at onset were strongly associated with increased risk of autoantibody loss for GADA (OR 29.9; *P* = 3.8 × 10^−14^), IA-2A (OR 72.8; *P* = 1.1 × 10^−14^), and ZnT8A (OR 95.9; *P* = 2.2 × 10^−16^) at final follow-up, independent of other covariates. For GADA (*P* = 3.8 × 10^−2^ to 1.5 × 10^−16^) and ZnT8A (*P* = 6.0 × 10^−5^ to 6.1 × 10^−18^), quartiles of baseline autoantibody titre below the highest quartile were strongly associated with increased risk of autoantibody loss; however, for IA-2A, discrimination of risk was only observed between the upper two and lower two quartiles (*P* = 2.8 × 10^−4^ to 1.1 × 10^−14^). Despite the heterogeneity between individuals, autoantibody titres decreased over follow-up across all quartiles of baseline titre. Interestingly, some individuals with lower quartiles of baseline titre still maintained autoantibody positivity ≥15 years diabetes duration and some individuals with higher quartiles of baseline titre rapidly lost autoantibody positivity within 5 years diabetes duration (Fig. 3). Importantly, the number of missing data points did not differ between the quartiles of titre at onset, suggesting that the influence of autoantibody titre on autoantibody loss at final follow-up did not result from skewed data.

### Age at onset

An older age-at-onset, considered as quartiles, and compared to individuals diagnosed <8 years, was negatively associated with autoantibody loss at final follow-up, but the pattern and strength of association were not uniform. For GADA, an age-at-onset ≥8years was negatively associated with GADA loss, with comparable odds across the upper age quartiles (average OR 0.4; *P* < 0.01–0.05). IA-2A loss was only less likely in individuals diagnosed between 11 and 14 years (OR 0.3; *P* = 0.012). In contrast, ZnT8A loss was less likely in individuals diagnosed ≥11 years (average OR 0.3; *P* = 0.002). These effects were independent of baseline titre but the influence of age at onset on both titres and longevity of humoral responses after onset is evident (Supplementary Fig. 6).

### Co-existing autoantibodies at onset

The presence of co-existing or the number of islet autoantibodies at onset was not associated with the loss of GADA, IA-2A, or ZnT8A at final sampling adjusted for non-genetic covariates (Supplementary Fig. 7). Compared with three autoantibodies at onset, the presence of two autoantibodies was weakly associated with ZnT8A loss at final follow-up (*P* = 0.045).

### Genetic associations of autoantibody positivity at initial sampling and autoantibody loss at final sampling

**Figure 4:**
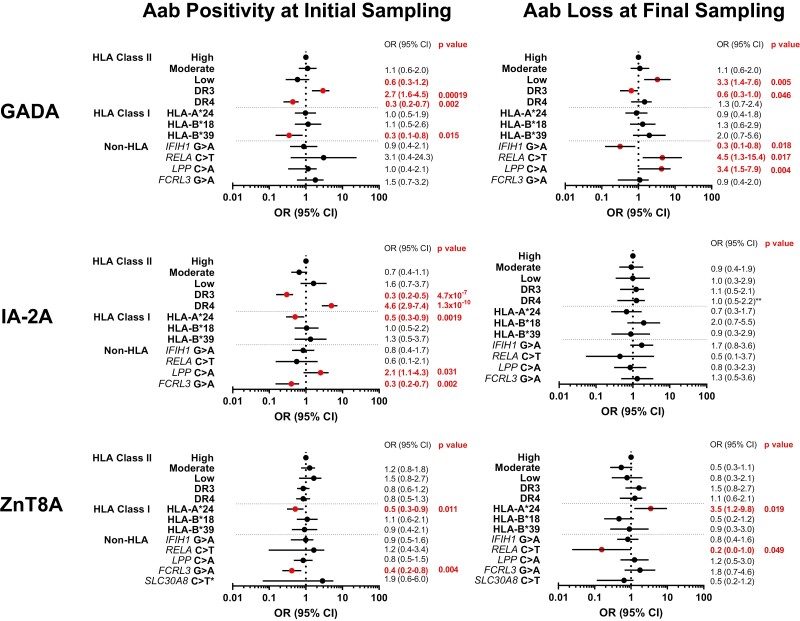
Genetic associations of autoantibody positivity around type 1 diabetes onset and autoantibody loss after onset. OR: odds ratio; 95% CI: confidence interval; OR of 1 denotes the reference category of variable; ORs over 1 favours autoantibody positivity at initial sampling (a) or autoantibody loss at final sampling (b); red dots and text denote alpha significance (<0.05). ORs, CIs and *P* values were calculated from multivariable logistic regression models adjusted for all non-genetic covariates [gender, age-at-onset, sampling time (months/years), and autoantibody titre at onset (final sampling only)]. All genetic covariates were considered independently in all models.

Multivariable models with complete genetic data for autoantibody positivity at initial sampling (<2 years from onset) and autoantibody loss at final sampling adjusted for non-genetic covariates (gender, age-at-onset, baseline autoantibody titre, and diabetes duration), genetic variables were considered independently as categorical or binary variables as previously stated (Fig. 4).

## GADA

At initial sampling, GADA positivity was not associated with HLA class II risk but was strongly associated with the presence of *DR3* (OR 2.7; *P* = 0.00019) and negatively with *DR4* (OR 0.3; *P* = 0.002) when considered independently. The presence of HLA class I HLA-B*39 was negatively associated with GADA positivity (OR 0.3; *P* = 0.015). Investigated non-HLA SNPs were not associated with GADA positivity at initial sampling. However, at final sampling, GADA loss was associated with low-risk HLA class II genotypes (OR 3.3; *P* = 0.005), possibly due to the reduced presence of at least one copy of DRB1*03. This was weakly confirmed by the negative association with *DR3* (OR 0.6; *P* = 0.046) when considered independently. Loss of GADA at final sampling was not associated with any HLA class I genotypes but was associated with 3 non-HLA SNPs; positively with the *RELA*/11q13 T allele (OR 4.5; *P* = 0.017) and *LPP*/3q28 A allele (OR 3.4; *P* = 0.004) but was negatively associated with the *IFIH1*/2q24 A allele (OR 0.3; *P* = 0.018).

## IA-2A

At initial sampling, IA-2A positivity was not associated with HLA class II genotypes but was strongly associated with the presence of *DR4* (OR 4.6; *P* = 1.3 × 10^−10^) and negatively with *DR3* (OR 0.3; *P* = 4.7 × 10^−7^) when considered independently. The presence of HLA class I HLA-A*24 was negatively associated with IA-2A positivity (OR 0.5; *P* = 0.0019). IA-2A positivity at initial sampling was also associated with two non-HLA SNPs; positively with the *LPP/*3q28 A allele (OR 2.1; *P* = 0.031) and negatively with the *FCRL3*/1q23 A allele (OR 0.3; *P* = 0.002). IA-2A loss at final sampling was not associated with any of the genotypes considered, independent of non-genetic covariates. However, the presence of at least one copy of *DR4* was negatively associated with IA-2A loss (OR 0.5; *P* = 0.016) when IA-2A titre at onset was excluded from the model due to an interaction between *DR4* and IA-2A titre (data not shown).

## ZnT8A

At initial sampling, ZnT8A positivity was not associated with any HLA class II genotypes but was negatively associated with the presence of HLA class I HLA-A*24 (OR 0.5; *P* = 0.011) and the non-HLA *FCRL3*/1q23 A allele (OR 0.4; *P* = 0.004). The *SLC30A8* genotype did not influence overall ZnT8A positivity but strongly influenced the specificity of the ZnT8A response (data not shown). The T allele was strongly associated with the development of ZnT8WA (OR 7.7; *P* = 5.8 × 10^−5^) and strongly negatively associated with the development of ZnT8RA (OR 0.1; *P* = 1.9 × 10^−7^). ZnT8A loss at final sampling was associated with the presence of HLA-A*24 (OR 3.5; *P* = 0.019). Only one non-HLA SNP showed a weak negative association with ZnT8A loss at final follow-up, the *RELA*/11q13 T allele (OR 0.2; *P* = 0.049).

## Discussion

In this study, we were able to analyse autoantibody loss (not just cross-sectional prevalence) because presence at diagnosis was established. The strongest predictor of islet autoantibody loss was lower baseline titres close to onset, while an age-at-onset under 8 years and a diabetes duration over 5 years were also associated. Genetic factors associated with autoantibody loss were antigen-specific and distinct from well-known associations with autoantibody positivity at onset. GADA loss was associated with low-risk HLA class II genotypes and non-HLA loci for *RELA*/11q13, *LPP*/3q28, and negatively with *IFIH1*/2q24. IA-2A loss was not linked with any genetic factors considered. ZnT8A loss was associated with the presence of HLA-A*24 and weakly with *RELA*/11q13. Despite an overall reduction in autoantibody prevalence, number, and titre as a function of increasing diabetes duration, the degree of autoantibody loss was not influenced by the number or combination of autoantibodies present at onset.

The strength of our study was the ability to account for baseline autoantibody positivity and titres, which we have shown, have the largest effect size with the persistence of humoral responses in the largest cohort of longitudinally sampled subjects with T1D. This allowed for the analysis of factors that may only be associated with autoantibody responses when baseline positivity and titre are accounted for, enabling the differentiation between prevalence and persistence of humoral responses after onset. A limitation of our current analysis was that the method (multivariable binary logistic regression) only utilised data from the last sample, instead of all available samples. We considered mixed-model effect generalised linear models (GLM) using a Restricted Maximum Likelihood fit and the Geisser–Greenhouse correction to estimate a linear trend accounting for missing values and sphericity in mean autoantibody levels over follow-up, respectively. When applied, this method suggested that there was evidence of a linear trend in decreasing autoantibody titres (*P* < 0.0001), but the within-subject variance of mean autoantibody titres was unequal across categories of longitudinal follow-up (Geisser-Greenhouse *ε* <0.5 in all responses). Similarly, the between-subject variance of mean autoantibody titres was high in all autoantibody responses (GADA SD: 240.3 DK units/ml; IA-2A SD: 83.0 DK units/ml; ZnT8A SD: 27.1 AU). Collectively, this indicated that autoantibody responses after onset are highly heterogeneous between individuals, and therefore, predictive modelling using linear trend or imputation would be inappropriate.

After onset (average median 7.3 years diabetes duration), only 40.1% remained positive for ZnT8A compared with, 70.8% and 76.8% for GADA and IA-2A, respectively. The rapid decline in ZnT8A, independent of R325W specificity during the first 10 years of diabetes, compared with GADA and IA-2A, has been reported previously [12, 13]. However, many studies have not been able to demonstrate the comparable persistence of both IA-2A and GADA, as generally prevalence of GADA is higher, particularly in older individuals [10]. Whilst it is difficult to compare between cross-sectional studies, where ZnT8A testing is often omitted, where GADA/IA-2A/ZnT8 was tested >10 years diabetes duration, there is agreement that autoantibody prevalence decreases with diabetes duration, but different frequencies of all biochemical autoantibodies were reported [12, 19, 20], and these studies were unable to account for baseline positivity status/titre. Increasingly islet autoantibody markers are being recommended and performed clinically to inform diabetes classification and insulin treatment, particularly in older individuals where type 2 diabetes is most prevalent [29–31]. Therefore, ZnT8A should be detected as close to diagnosis as possible but GADA and IA-2A can be detected for many years in the majority where they were positive at diagnosis. This could inform post-diagnosis diabetes classification in adults.

Measuring islet autoantibodies after diagnosis may also provide important insights into the mechanisms driving ongoing islet autoimmunity and loss of beta-cell function after onset. The results of the present study into autoantibody patterns in addition to, non-genetic and genetic factors may help elucidate these mechanisms. For instance, islet autoantibody loss was strongly influenced by titre around onset but analysis of longitudinal humoral titres after onset showed that periodic enhancement of titres is rare; titres of GADA, IA-2A, and ZnT8A progressively decreased over follow-up in 74.9%, 90.0%, and 98.5%, respectively. This suggests that islet autoantibody responses are predominantly governed by immune memory. However, for GADA our study supports previous reports that GADA titres can peak or stabilize after diagnosis in select individuals, but this may be a distinct feature of GADA responses and the mechanism is not clear [14, 32, 33]. Previously, one longitudinal study tested subsets of samples taken at 1-13 years diabetes duration in 116 subjects from onset (aged <18 years) in the Czech Republic and found a seroconversion frequency of 6–8% after diagnosis [16]. However, a limitation of our study is that we cannot comment on possible re-emergence of additional autoantibody seroconversion because sequential samples after the loss of autoantibody positivity were not tested. Collectively, current data indicate that antigen spreading of humoral responses occurs predominantly during the prodrome of T1D and is unlikely to be a strong feature of ongoing humoral autoimmunity towards residual beta-cells.

Islet autoantibody positivity at diabetes onset has been linked to age at diagnosis which we have confirmed in this cohort [11, 14, 34, 35] but few studies have been able to investigate the influence of age at diagnosis on the persistence of humoral responses. In this study, we found that loss of GADA, IA-2A, and ZnT8A is slower in older individuals when corrected for diabetes duration, and importantly, autoantibody titre at diagnosis. Other smaller studies with multivariable analysis have also observed this for GADA and IA-2A [14, 33]. Age-associated autoantibody positivity profiles during diabetes may in part be related to histologically distinct T1D endotypes recently proposed (<7 years and ≥13 years) [36], but regardless of how age was considered (endotype versus quartile of age) in multivariable analysis, a younger age at onset was associated with loss of autoantibody responses in this cohort (data not shown). Whilst this cohort included some cases of adult T1D, the majority of the cohort were under 20 years old at diagnosis which limits our exploration of this factor. It is clear that age is an important factor in the heterogeneity of T1D and both the development and longevity of islet autoimmunity remain to be fully characterized in adults at all stages of diabetes. Of the non-genetic factors assessed, only autoantibody titre and age at onset were associated with loss of humoral responses. Whether titre or age at diagnosis is related to beta-cell mass or development of immunological memory and senescence, requires future study.

Next, we asked ‘do the genetic drivers of autoantibody production after onset differ from the well-known antigen-specific variants that influence T1D progression and risk?’ For this, we specifically chose to analyse genotypes and genetic loci that have been associated with islet autoantibody positivity before, around, and after diagnosis of T1D. The highly predisposing HLA class II genotypes for T1D risk that strongly influence titres of specific autoantibodies at onset (especially IA-2A and IAA) do not have additional effects on humoral responses after clinical onset. Previous studies investigating prevalence after diagnosis have shown an association of GADA with DRB1*03 and IA-2A with DRB1*04 [11, 35, 37, 38]; however, were unable to correct for autoantibody level at diagnosis. In contrast, we found a weak negative association between DRB1*03 and GADA loss, a positive association between low HLA class II risk and GADA loss, and DRB1*04 was only negatively associated with IA-2A loss when baseline titre was not considered. Type 1 diabetes-associated HLA class I genotypes were not associated with loss of GADA or IA-2A, but the presence of HLA-A*24 was associated with loss of ZnT8A in adjusted models. This is in line with the negative association between HLA-A*24 and ZnT8A positivity which we have confirmed and have previously reported at onset [39] and in first-degree relatives [40], which may suggest that attenuation of humoral responses to ZnT8A in HLA-A*24 carriers continues after diabetes onset. A previous study has shown a negative association of HLA-A*24 with IA-2A positivity after diagnosis but was unable to account for IA-2A status at onset [34]. We only observed this effect at onset as previously published in an overlapping BOX study cohort [39]. An association between HLA-B*39 and progression of autoimmunity has been previously reported [41], but the negative association identified between HLA-B*39 and GADA positivity at onset was not observed after onset, suggesting distinct effects of these HLA class I genotypes on GADA and IA-2A responses around onset and after diagnosis.

The SNPs investigated in this study and/or genetic loci have also been linked with positivity for specific humoral responses 3–14 years after T1D onset in ~7000 individuals from the T1D genetic consortium (aged <17 years; median age at onset 8 years) [11, 27]. In age-at-onset and diabetes duration adjusted models, *RELA/*11q13 and *FCRL3*/1q23 SNPs were associated with IA-2A positivity, *LPP*/3q28 SNPs were associated with GADA positivity, and *IFIH1*/2q24 was associated with positivity for autoantibodies related to autoimmune gastritis and thyroid disease [11]. In a separate analysis of overlapping populations, *FCRL3*/1q23 was negatively associated with IA-2A and ZnT8A positivity sampled <2 years of onset [42, 43], which we replicated for ZnT8A but not IA-2A. In contrast, we found that *RELA*/11q13 and *LPP*/3q28 were associated with GADA loss, but no SNPs studied were associated with IA-2A loss, the minor allele of *IFIH1*/2q24 (major allele confers risk for diabetes) was negatively associated with GADA loss, and *RELA*/11q13 was weakly negatively associated with ZnT8A loss. Despite the size difference in both cohorts (577 vs ~7000), the different effects of *RELA*/11q13, *LPP*/3q28, and *IFIH1*/2q24 may be related to the present study having a longer follow-up period and correction for baseline autoantibody titre rather than statistical power. Of all the SNPs we studied, only *IFIH1/*2q24 (rs2111485 in LD with rs1990760) has been associated with both T1D risk [44] and progression to T1D [45], whereas, the others have been linked to other autoimmune conditions [46–48]. A limitation of our study is that we did not have sufficient power to analyse all T1D-associated SNPs. These SNPs and/or genetic loci have previously been associated with non-islet autoimmunity, which we and others have shown are common in individuals with type 1 diabetes [49]. Overall, most HLA and non-HLA genetic factors influencing the prevalence of autoantibodies at diagnosis were not associated with autoantibody loss, suggesting that the processes driving autoantibody development before diagnosis do not continue to drive the maintenance of autoantibody levels/responses after diagnosis.

Studies investigating long-duration (>20–50 years) diabetes suggest some individuals have residual beta-cells with the functional capacity to secrete insulin (C-peptide) [18, 50]. Recent reports have suggested that incorporating autoantibody titre may improve predictive models for C-peptide and clinical characteristics after diagnosis [51, 52]. An older age at onset has also been associated with the less rapid decline of C-peptide [21, 53], which may partially explain why older individuals had a less rapid loss of humoral responses in the present study. However, previous BOX data and other studies suggest autoantibody positivity at and after onset did not strongly correlate with C-peptide in longstanding cases [12, 15, 17, 18]. We had few additional data from BOX participants and therefore, this was not re-analysed here but sample collection is ongoing.

If residual antigen remains accessible to immune surveillance either by continued beta-cell death or functional residual beta-cells, the differential antigen expression may provide some rationale as to why ZnT8A is lost more rapidly than GADA or IA-2A. All islet autoantigens are highly expressed in pancreatic islets but ZnT8 expression is almost exclusive to beta-cells and is exposed upon glucose-stimulated insulin secretion whereas, GAD and IA-2 are found in specific cells of the nervous system [54]. However, considering the findings from present and previous studies, it seems unlikely that the presence of residual beta-cells is the only factor driving sustained islet autoantibody production. There are tissue-resident long-lived plasma cells (LLPCs) that form an independent compartment of immunological memory that may be involved in long-duration T1D [55]. LLPCs can persist for decades independent of B lymphocyte precursors or residual antigens but are not intrinsically long-lived. Their survival is dependent on specialized niche microenvironments, but the cellular and molecular components that promote LLPC production or survival are not fully characterized in humans; there is some evidence in other autoimmune diseases of LLPCs in inflamed target tissue [55–57]. Could these be responsible for residual autoantibody production in some individuals decades after diagnosis?

The clinical usefulness of islet autoantibodies after diagnosis is often questioned but the data from this study present the longest duration of longitudinal autoantibody analysis thus far. Where islet autoantibodies are used to help define diabetes aetiology, testing close to diagnosis is important particularly for ZnT8A, as responses are rapidly lost. This study also suggests that the drivers of islet autoantibody prevalence before diagnosis and persistence after diagnosis are distinct. Whilst islet autoantibody loss is heterogeneous, factors associated with loss are titre, diabetes duration, and age at diagnosis, select HLA Class I/II alleles and non-HLA SNPs. These factors may also be important to consider when interpreting the impact of immunotherapy on islet autoantibodies after the onset of T1D, and what this indicates about the mechanistic actions of the therapy. Therefore, the meaning of islet autoantibodies after T1D onset, merits further investigation, because it has implications for clinical trials and understanding of the ongoing islet autoimmunity after diagnosis in some individuals.

## Supplementary Material

uxac087_suppl_Supplementary_Material

## Data Availability

The datasets are available from the corresponding author on reasonable request.

## Abbreviations

BOX: Bart’s Oxford family study
GADA: glutamate decarboxylase 65 autoantibodies
GLM: generalised linear models
IAA: insulin autoantibodies
IA-2A: islet antigen 2 autoantibodies
LLPC: long-lived plasma cells
T1D: type 1 diabetes
ZnT8A: zinc transporter 8 autoantibodies
ZnT8RA: zinc transporter 8 arginine-specific autoantibodies
ZnT8WA: zinc transporter 8 tryptophan-specific autoantibodies

## Permission to reproduce

Not Applicable. All content is the original work of the authors.
