# Supplementary material for: The longitudinal loss of islet autoantibody responses from diagnosis of type 1 diabetes occurs progressively over follow-up and is determined by low autoantibody titres, early-onset, and genetic variants
Source: Clin Exp Immunol. 2022 Oct 1;210(2):151–62. doi: 10.1093/cei/uxac087 (PMC9750828; doi:10.1093/cei/uxac087)
Supplement: uxac087_suppl_Supplementary_Material [file uxac087_suppl_supplementary_material.ppt]

## Slide 1
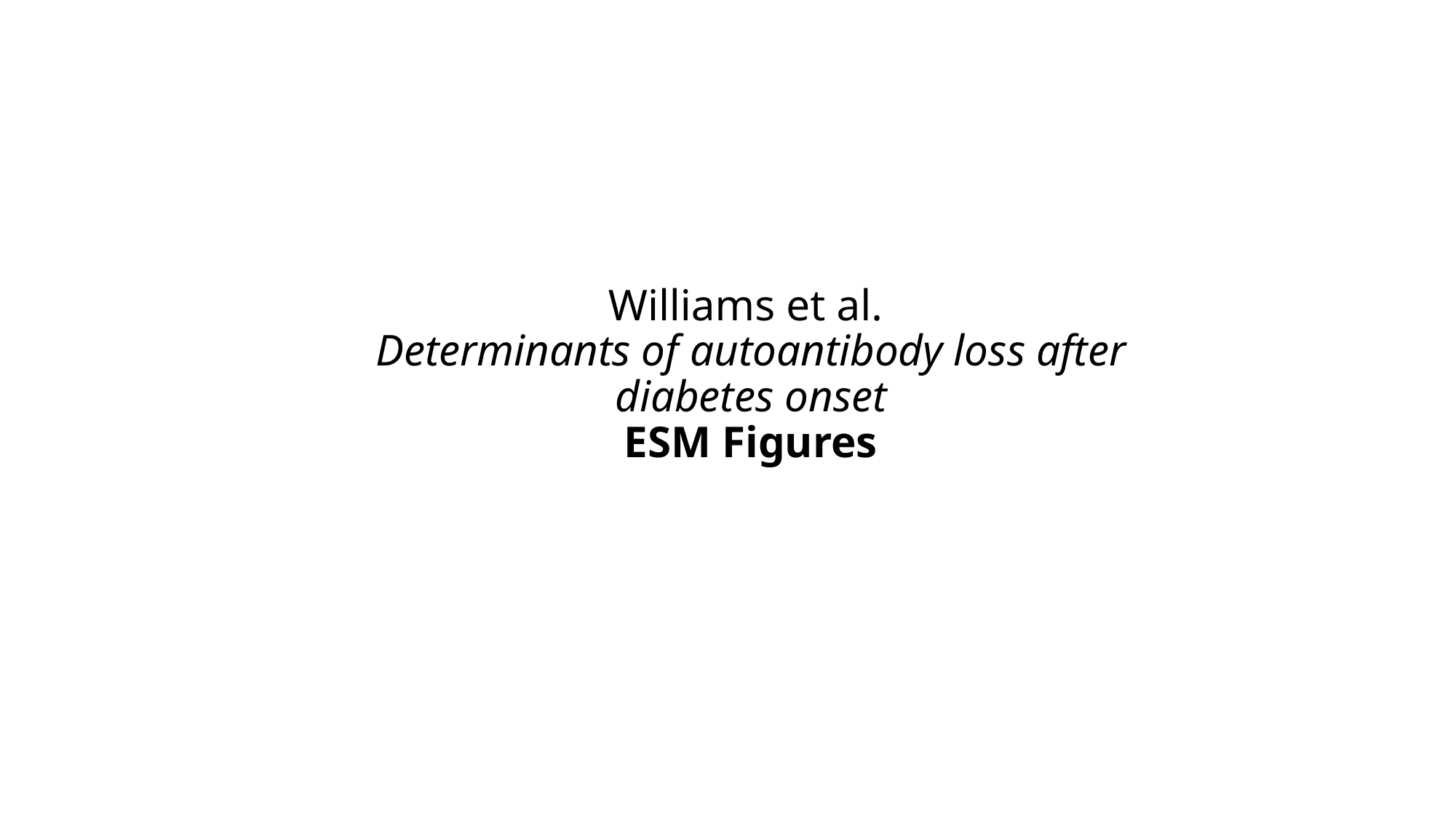

# Williams et al. Determinants of autoantibody loss after diabetes onset ESM Figures

## Slide 2
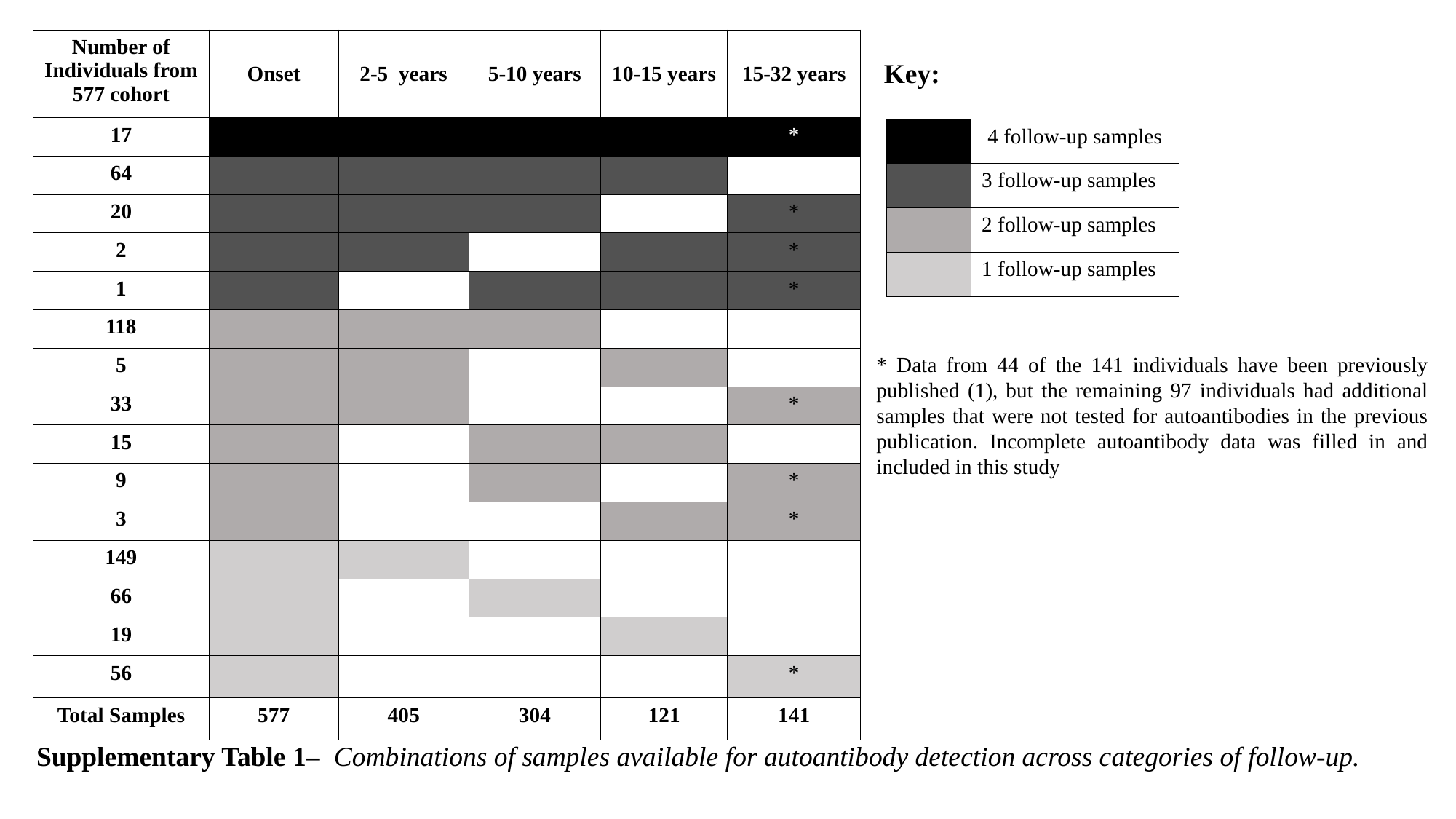

| Number of Individuals from 577 cohort | Onset | 2-5 years | 5-10 years | 10-15 years | 15-32 years |
| --- | --- | --- | --- | --- | --- |
| 17 | | | | | \* |
| 64 | | | | | |
| 20 | | | | | \* |
| 2 | | | | | \* |
| 1 | | | | | \* |
| 118 | | | | | |
| 5 | | | | | |
| 33 | | | | | \* |
| 15 | | | | | |
| 9 | | | | | \* |
| 3 | | | | | \* |
| 149 | | | | | |
| 66 | | | | | |
| 19 | | | | | |
| 56 | | | | | \* |
| Total Samples | 577 | 405 | 304 | 121 | 141 |
Key:
| | 4 follow-up samples |
| --- | --- |
| | 3 follow-up samples |
| | 2 follow-up samples |
| | 1 follow-up samples |
* Data from 44 of the 141 individuals have been previously published (1), but the remaining 97 individuals had additional samples that were not tested for autoantibodies in the previous publication. Incomplete autoantibody data was filled in and included in this study
Supplementary Table 1– Combinations of samples available for autoantibody detection across categories of follow-up.

## Slide 3
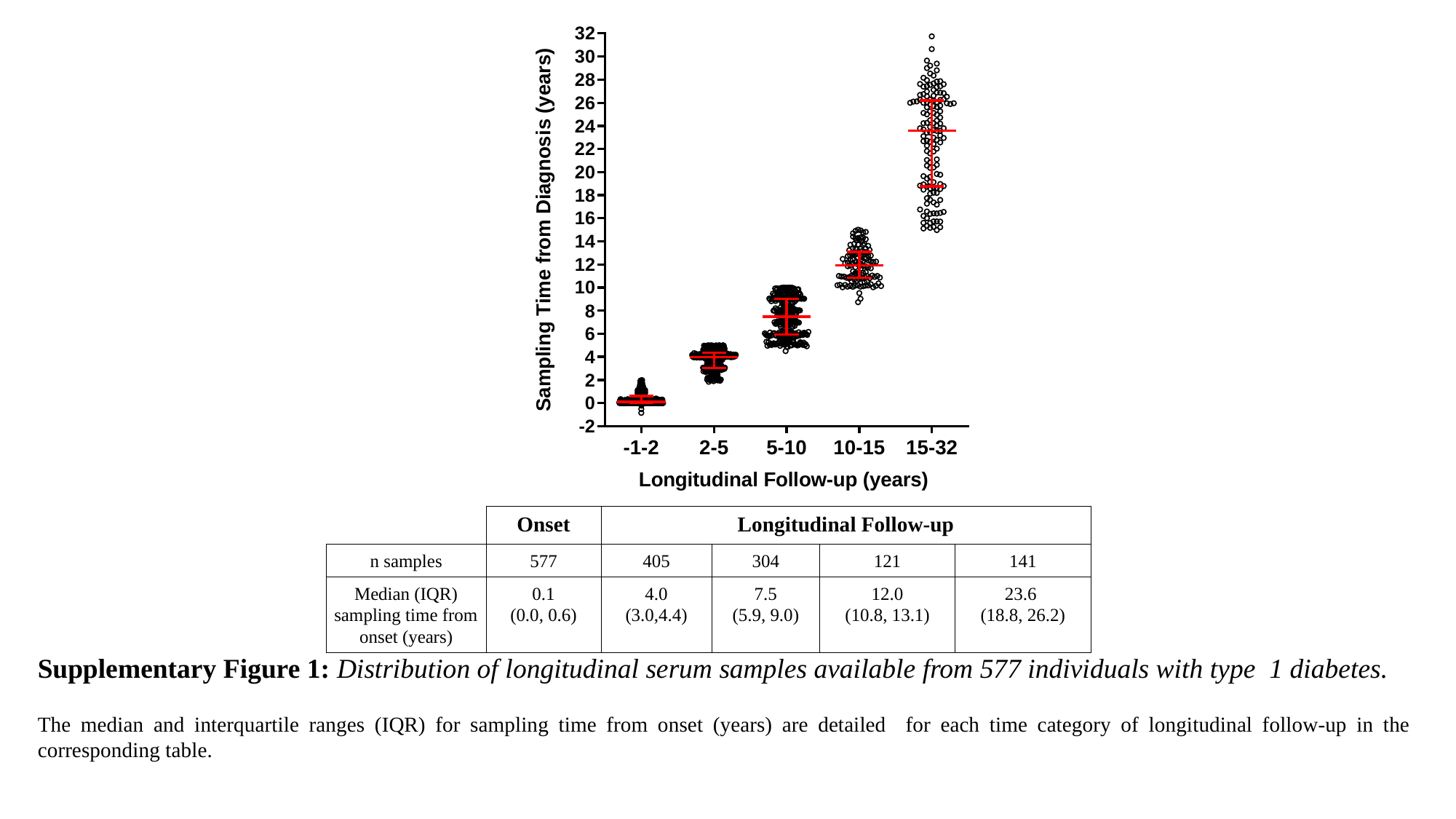

| | Onset | Longitudinal Follow-up | | | |
| --- | --- | --- | --- | --- | --- |
| n samples | 577 | 405 | 304 | 121 | 141 |
| Median (IQR) sampling time from onset (years) | 0.1 (0.0, 0.6) | 4.0 (3.0,4.4) | 7.5 (5.9, 9.0) | 12.0 (10.8, 13.1) | 23.6 (18.8, 26.2) |
Supplementary Figure 1: Distribution of longitudinal serum samples available from 577 individuals with type 1 diabetes.
The median and interquartile ranges (IQR) for sampling time from onset (years) are detailed for each time category of longitudinal follow-up in the corresponding table.

## Slide 4
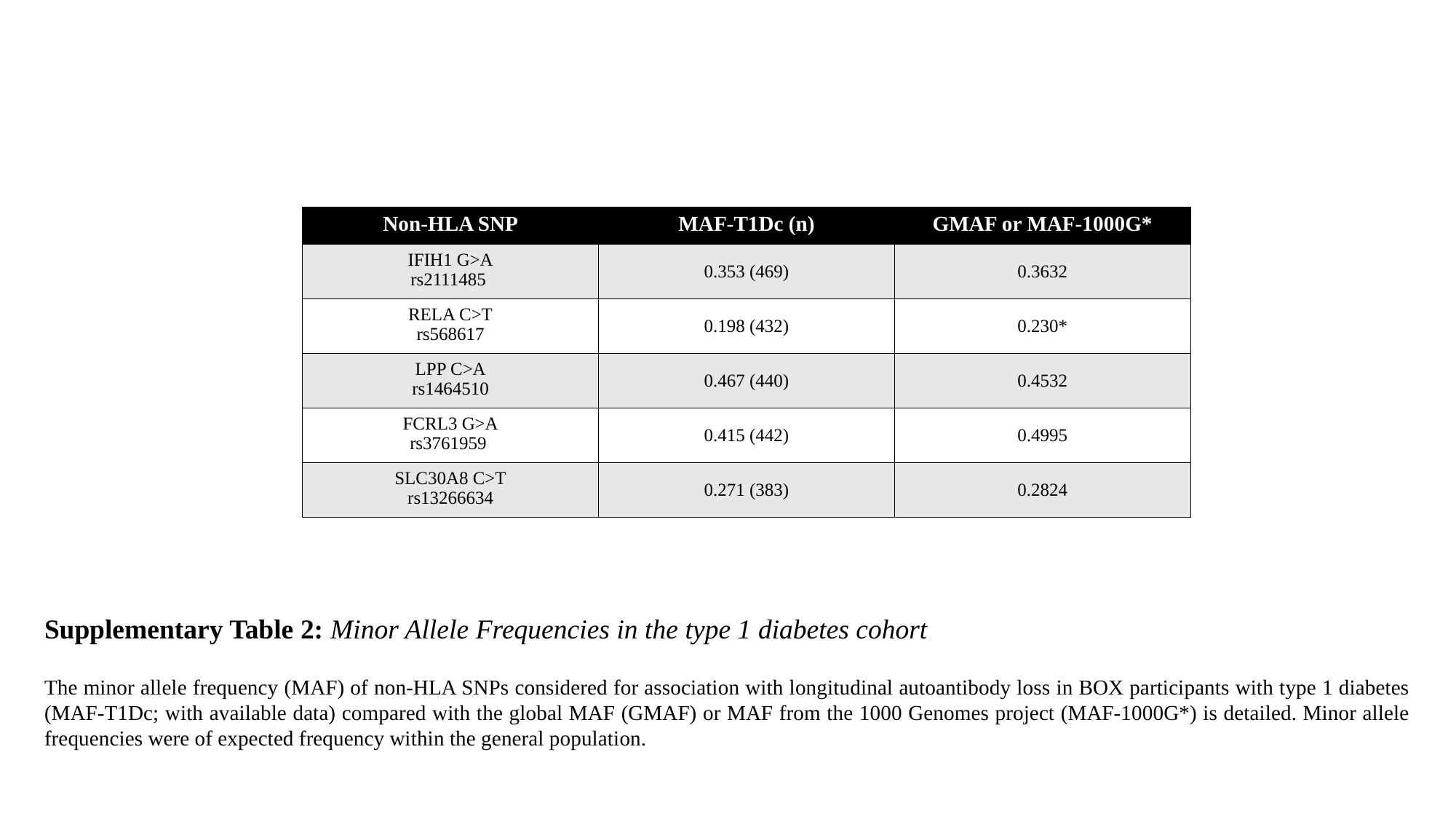

| Non-HLA SNP | MAF-T1Dc (n) | GMAF or MAF-1000G\* |
| --- | --- | --- |
| IFIH1 G>A rs2111485 | 0.353 (469) | 0.3632 |
| RELA C>T rs568617 | 0.198 (432) | 0.230\* |
| LPP C>A rs1464510 | 0.467 (440) | 0.4532 |
| FCRL3 G>A rs3761959 | 0.415 (442) | 0.4995 |
| SLC30A8 C>T rs13266634 | 0.271 (383) | 0.2824 |
Supplementary Table 2: Minor Allele Frequencies in the type 1 diabetes cohort
The minor allele frequency (MAF) of non-HLA SNPs considered for association with longitudinal autoantibody loss in BOX participants with type 1 diabetes (MAF-T1Dc; with available data) compared with the global MAF (GMAF) or MAF from the 1000 Genomes project (MAF-1000G*) is detailed. Minor allele frequencies were of expected frequency within the general population.

## Slide 5
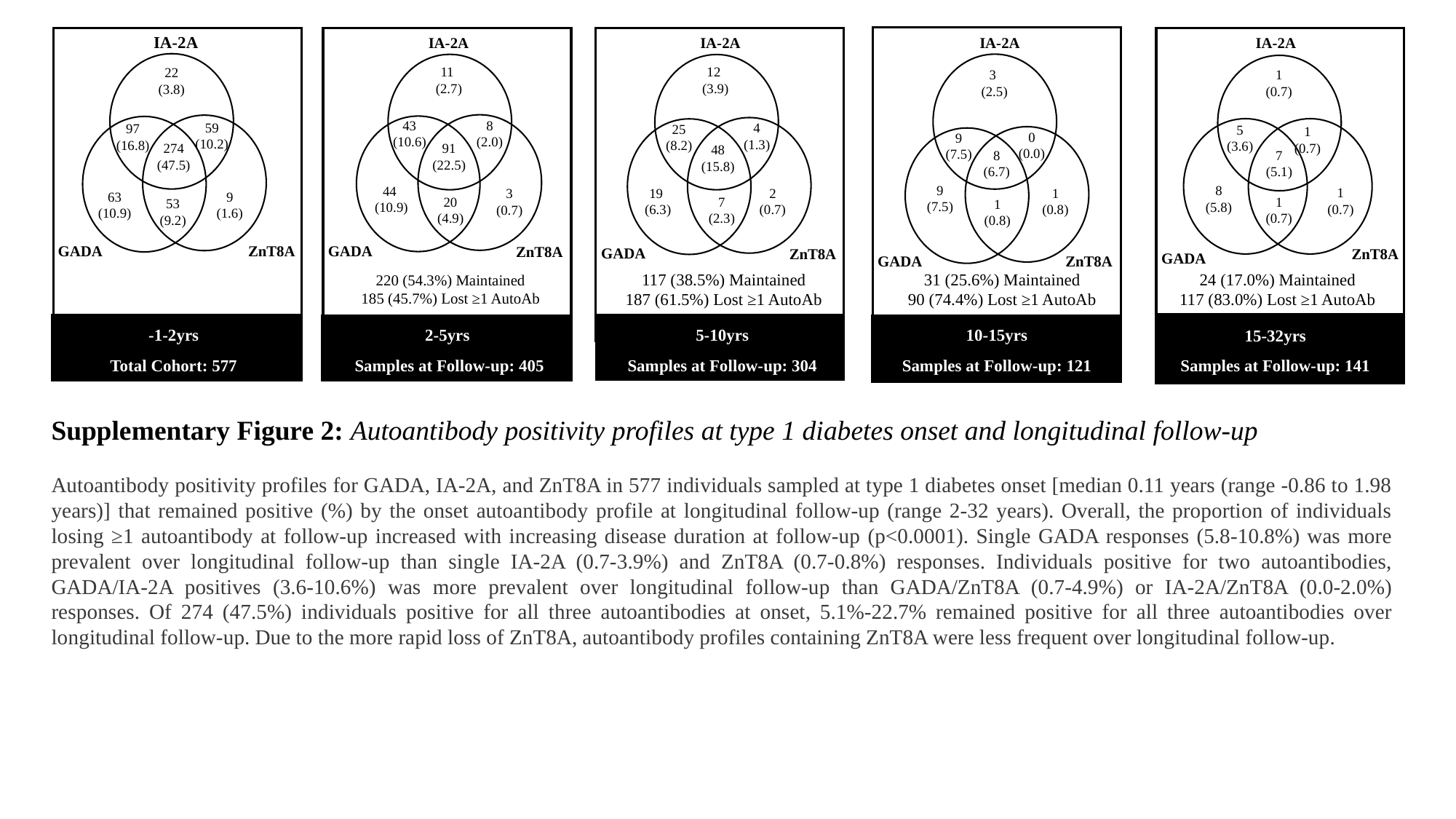

IA-2A
22
(3.8)
59
(10.2)
97
(16.8)
274
(47.5)
63
(10.9)
9
(1.6)
53
(9.2)
GADA
ZnT8A
IA-2A
11
(2.7)
43
(10.6)
8
(2.0)
91
(22.5)
44
(10.9)
3
(0.7)
20
(4.9)
GADA
ZnT8A
IA-2A
12
(3.9)
4
(1.3)
25
(8.2)
48
(15.8)
19
 (6.3)
2
(0.7)
7
(2.3)
GADA
ZnT8A
IA-2A
3
(2.5)
0
(0.0)
9
(7.5)
8
(6.7)
9
(7.5)
1
(0.8)
1
(0.8)
GADA
ZnT8A
IA-2A
1
(0.7)
5
(3.6)
1
(0.7)
7
(5.1)
8
(5.8)
1
(0.7)
1
(0.7)
ZnT8A
GADA
117 (38.5%) Maintained
187 (61.5%) Lost ≥1 AutoAb
31 (25.6%) Maintained
90 (74.4%) Lost ≥1 AutoAb
220 (54.3%) Maintained
185 (45.7%) Lost ≥1 AutoAb
-1-2yrs
Total Cohort: 577
2-5yrs
Samples at Follow-up: 405
5-10yrs
Samples at Follow-up: 304
10-15yrs
Samples at Follow-up: 121
15-32yrs
Samples at Follow-up: 141
24 (17.0%) Maintained
117 (83.0%) Lost ≥1 AutoAb
Supplementary Figure 2: Autoantibody positivity profiles at type 1 diabetes onset and longitudinal follow-up
Autoantibody positivity profiles for GADA, IA-2A, and ZnT8A in 577 individuals sampled at type 1 diabetes onset [median 0.11 years (range -0.86 to 1.98 years)] that remained positive (%) by the onset autoantibody profile at longitudinal follow-up (range 2-32 years). Overall, the proportion of individuals losing ≥1 autoantibody at follow-up increased with increasing disease duration at follow-up (p<0.0001). Single GADA responses (5.8-10.8%) was more prevalent over longitudinal follow-up than single IA-2A (0.7-3.9%) and ZnT8A (0.7-0.8%) responses. Individuals positive for two autoantibodies, GADA/IA-2A positives (3.6-10.6%) was more prevalent over longitudinal follow-up than GADA/ZnT8A (0.7-4.9%) or IA-2A/ZnT8A (0.0-2.0%) responses. Of 274 (47.5%) individuals positive for all three autoantibodies at onset, 5.1%-22.7% remained positive for all three autoantibodies over longitudinal follow-up. Due to the more rapid loss of ZnT8A, autoantibody profiles containing ZnT8A were less frequent over longitudinal follow-up.

## Slide 6
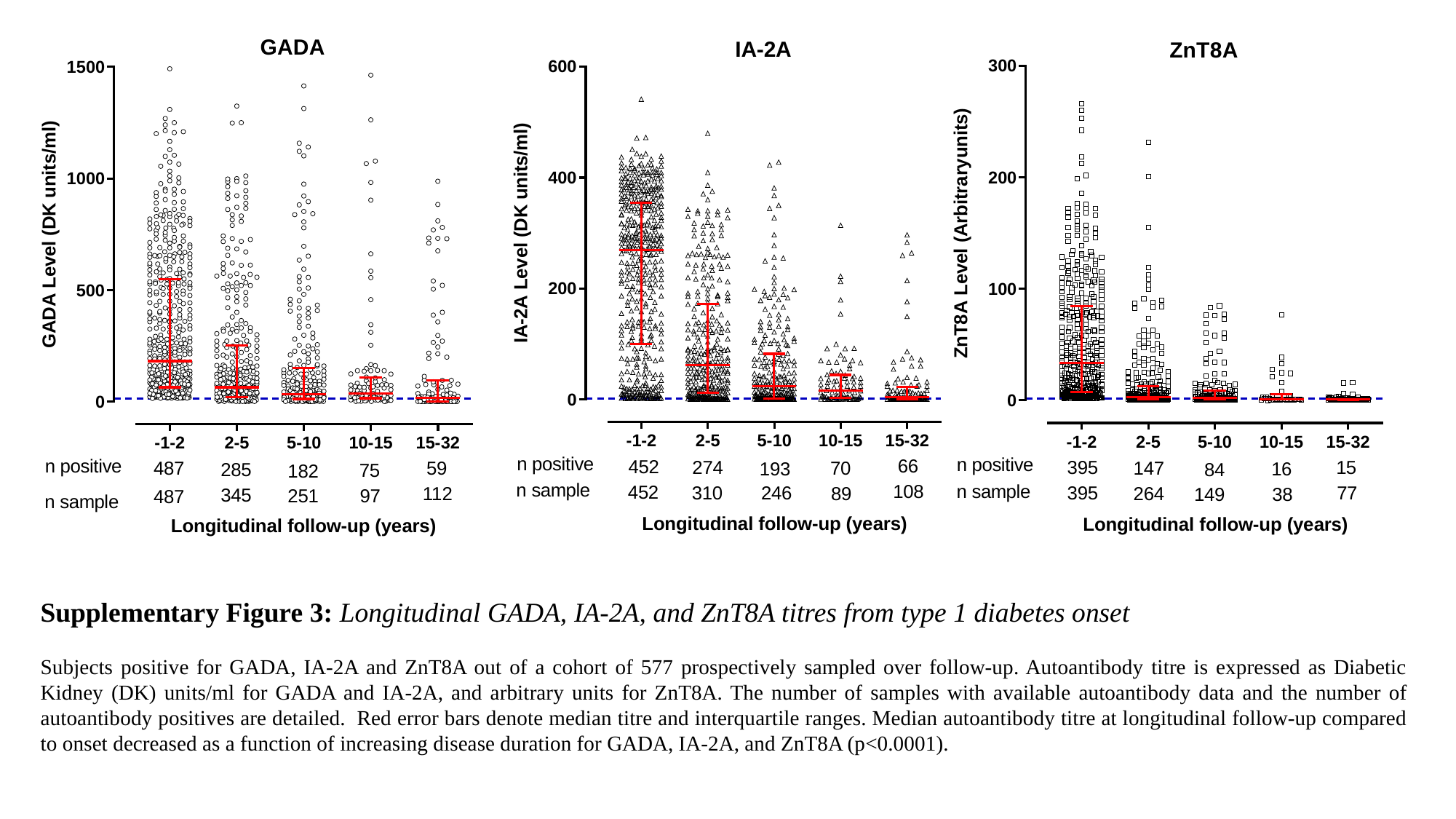

Supplementary Figure 3: Longitudinal GADA, IA-2A, and ZnT8A titres from type 1 diabetes onset
Subjects positive for GADA, IA-2A and ZnT8A out of a cohort of 577 prospectively sampled over follow-up. Autoantibody titre is expressed as Diabetic Kidney (DK) units/ml for GADA and IA-2A, and arbitrary units for ZnT8A. The number of samples with available autoantibody data and the number of autoantibody positives are detailed. Red error bars denote median titre and interquartile ranges. Median autoantibody titre at longitudinal follow-up compared to onset decreased as a function of increasing disease duration for GADA, IA-2A, and ZnT8A (p<0.0001).

## Slide 7
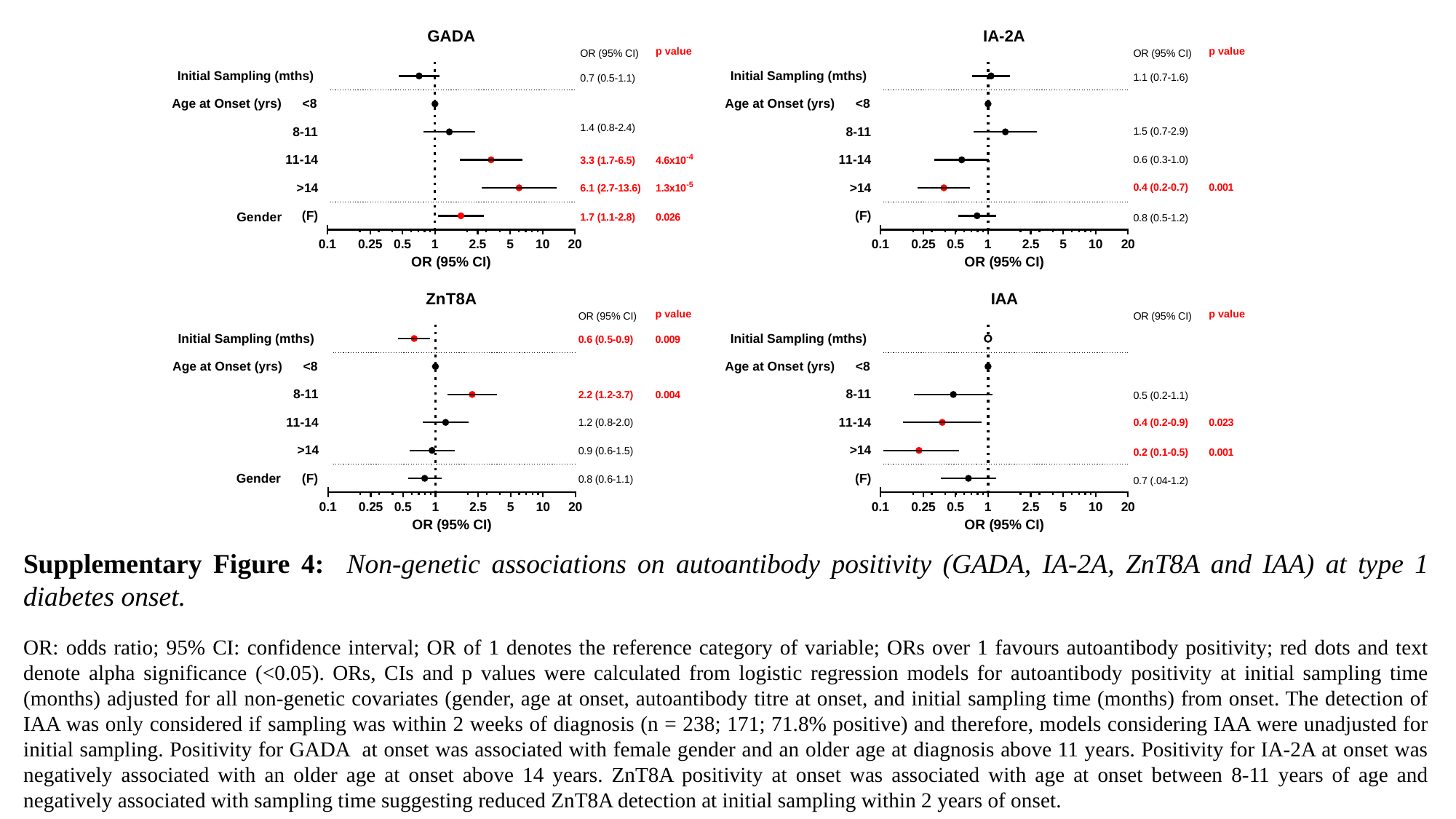

Supplementary Figure 4: Non-genetic associations on autoantibody positivity (GADA, IA-2A, ZnT8A and IAA) at type 1 diabetes onset.
OR: odds ratio; 95% CI: confidence interval; OR of 1 denotes the reference category of variable; ORs over 1 favours autoantibody positivity; red dots and text denote alpha significance (<0.05). ORs, CIs and p values were calculated from logistic regression models for autoantibody positivity at initial sampling time (months) adjusted for all non-genetic covariates (gender, age at onset, autoantibody titre at onset, and initial sampling time (months) from onset. The detection of IAA was only considered if sampling was within 2 weeks of diagnosis (n = 238; 171; 71.8% positive) and therefore, models considering IAA were unadjusted for initial sampling. Positivity for GADA at onset was associated with female gender and an older age at diagnosis above 11 years. Positivity for IA-2A at onset was negatively associated with an older age at onset above 14 years. ZnT8A positivity at onset was associated with age at onset between 8-11 years of age and negatively associated with sampling time suggesting reduced ZnT8A detection at initial sampling within 2 years of onset.

## Slide 8
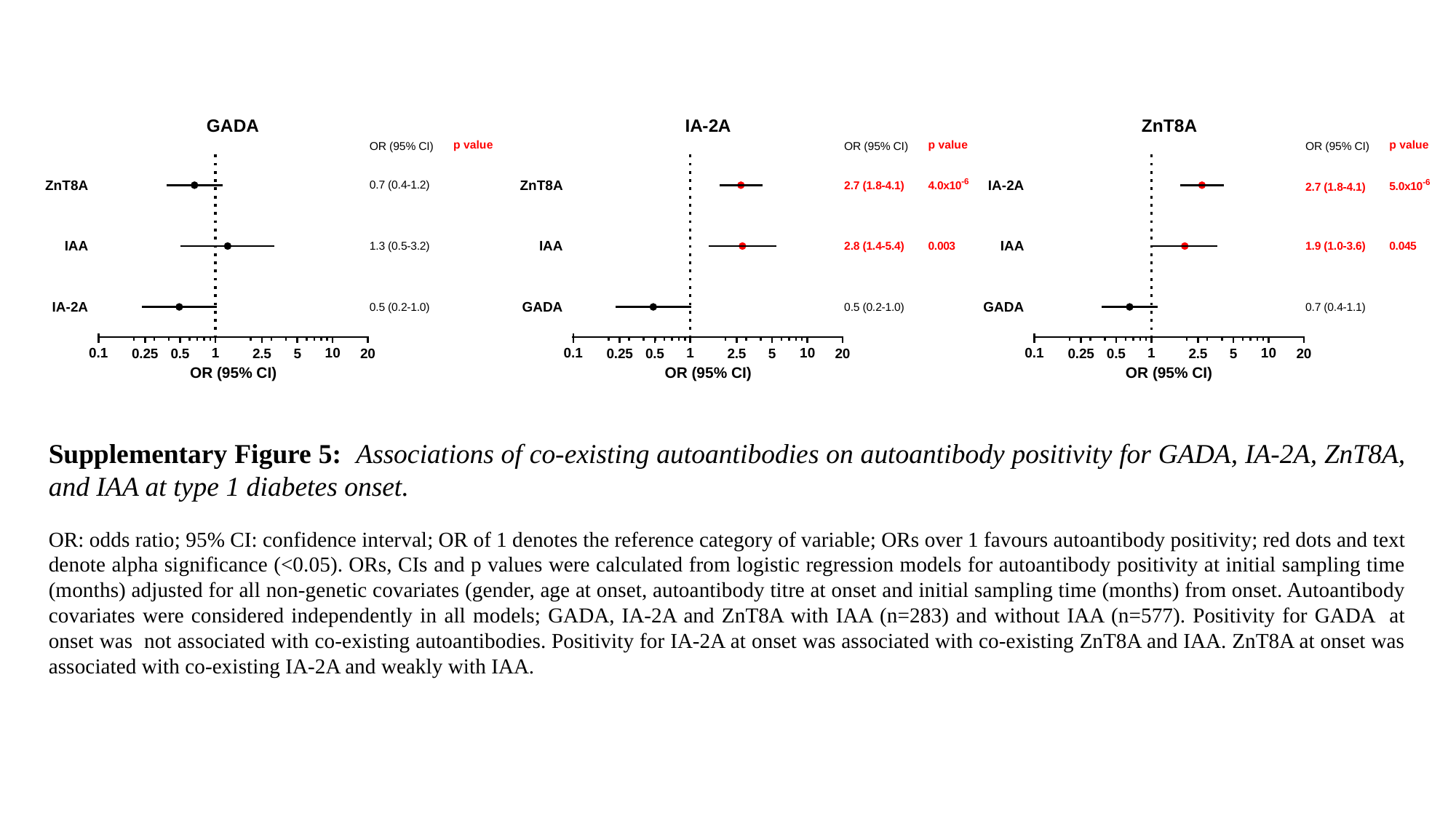

Supplementary Figure 5: Associations of co-existing autoantibodies on autoantibody positivity for GADA, IA-2A, ZnT8A, and IAA at type 1 diabetes onset.
OR: odds ratio; 95% CI: confidence interval; OR of 1 denotes the reference category of variable; ORs over 1 favours autoantibody positivity; red dots and text denote alpha significance (<0.05). ORs, CIs and p values were calculated from logistic regression models for autoantibody positivity at initial sampling time (months) adjusted for all non-genetic covariates (gender, age at onset, autoantibody titre at onset and initial sampling time (months) from onset. Autoantibody covariates were considered independently in all models; GADA, IA-2A and ZnT8A with IAA (n=283) and without IAA (n=577). Positivity for GADA at onset was not associated with co-existing autoantibodies. Positivity for IA-2A at onset was associated with co-existing ZnT8A and IAA. ZnT8A at onset was associated with co-existing IA-2A and weakly with IAA.

## Slide 9
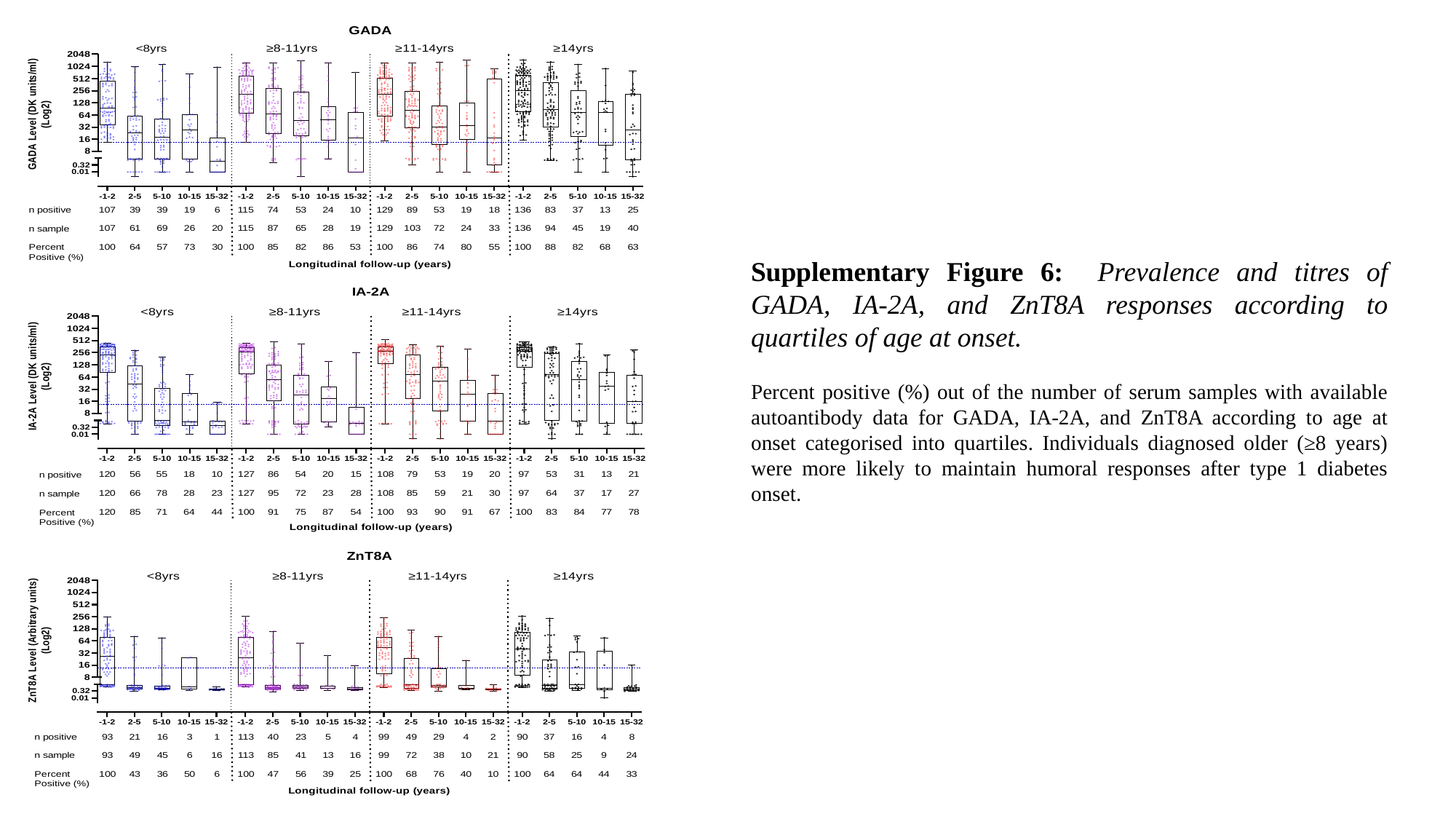

Supplementary Figure 6: Prevalence and titres of GADA, IA-2A, and ZnT8A responses according to quartiles of age at onset.
Percent positive (%) out of the number of serum samples with available autoantibody data for GADA, IA-2A, and ZnT8A according to age at onset categorised into quartiles. Individuals diagnosed older (≥8 years) were more likely to maintain humoral responses after type 1 diabetes onset.

## Slide 10
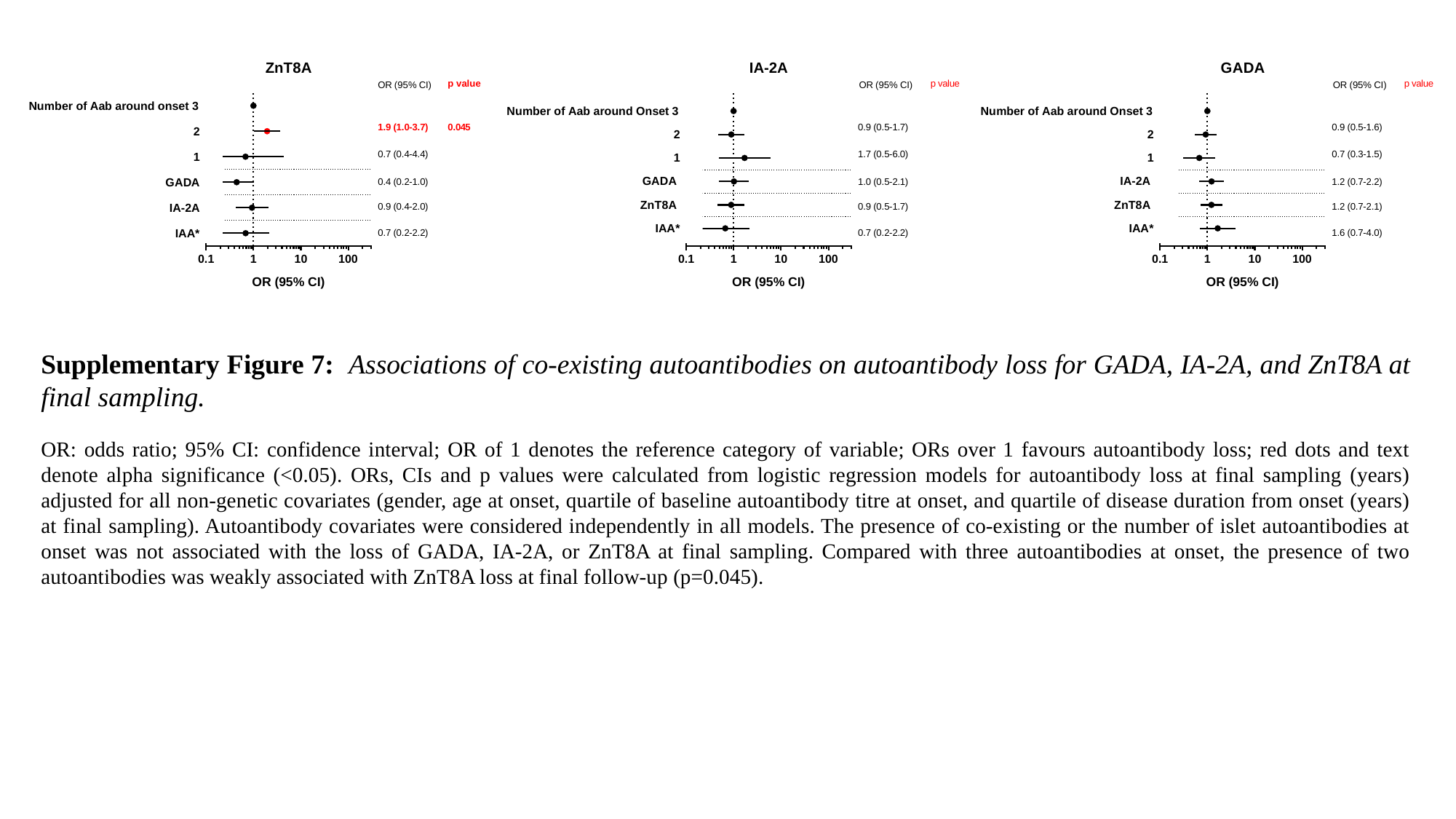

Supplementary Figure 7: Associations of co-existing autoantibodies on autoantibody loss for GADA, IA-2A, and ZnT8A at final sampling.
OR: odds ratio; 95% CI: confidence interval; OR of 1 denotes the reference category of variable; ORs over 1 favours autoantibody loss; red dots and text denote alpha significance (<0.05). ORs, CIs and p values were calculated from logistic regression models for autoantibody loss at final sampling (years) adjusted for all non-genetic covariates (gender, age at onset, quartile of baseline autoantibody titre at onset, and quartile of disease duration from onset (years) at final sampling). Autoantibody covariates were considered independently in all models. The presence of co-existing or the number of islet autoantibodies at onset was not associated with the loss of GADA, IA-2A, or ZnT8A at final sampling. Compared with three autoantibodies at onset, the presence of two autoantibodies was weakly associated with ZnT8A loss at final follow-up (p=0.045).

## Slide 11
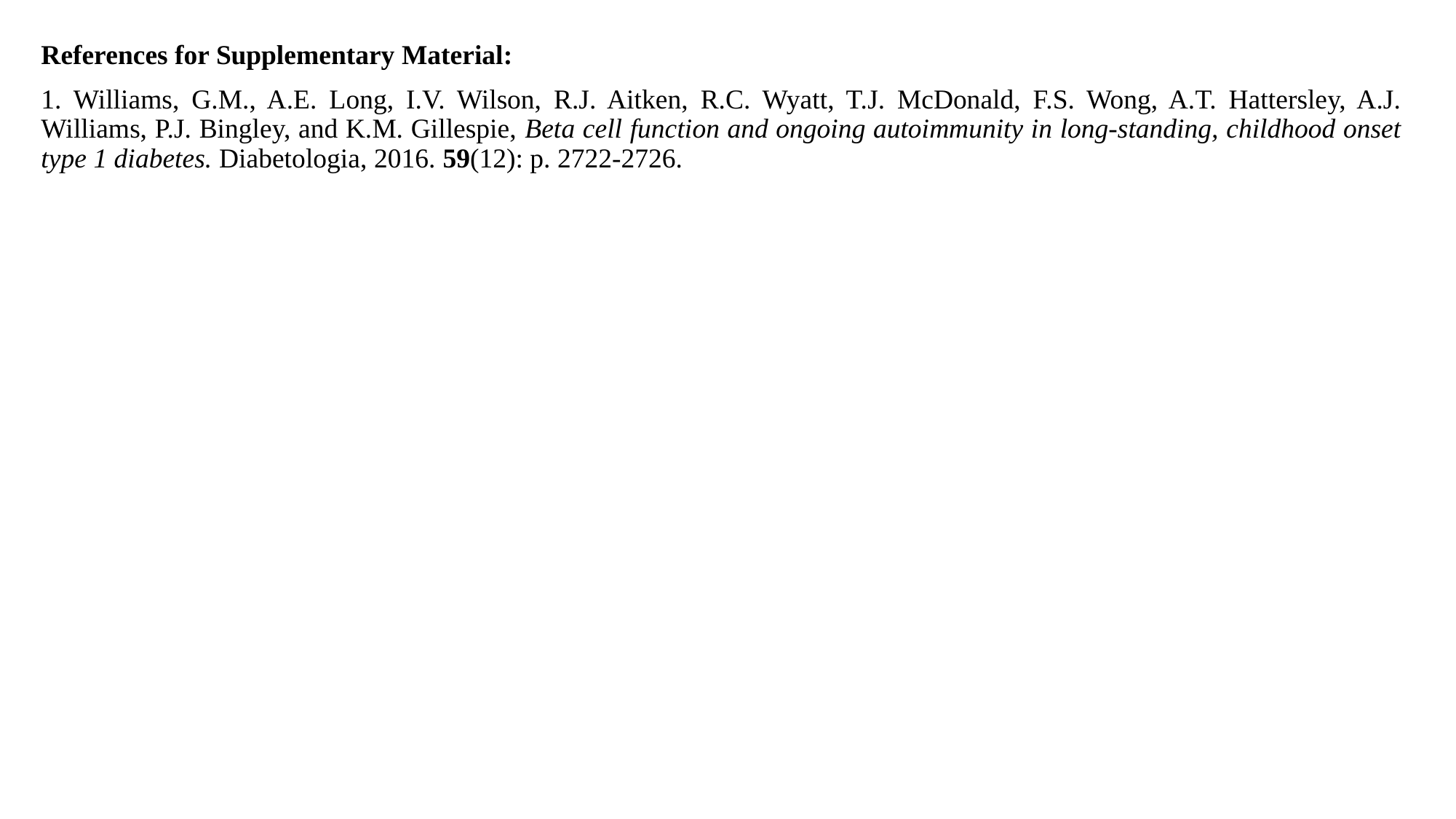

References for Supplementary Material:
1. Williams, G.M., A.E. Long, I.V. Wilson, R.J. Aitken, R.C. Wyatt, T.J. McDonald, F.S. Wong, A.T. Hattersley, A.J. Williams, P.J. Bingley, and K.M. Gillespie, Beta cell function and ongoing autoimmunity in long-standing, childhood onset type 1 diabetes. Diabetologia, 2016. 59(12): p. 2722-2726.
